# The sad tale of a youthful right ventricle struggling with endomyocardial fibrosis

**DOI:** 10.1093/omcr/omad158

**Published:** 2024-02-16

**Authors:** Samah El-Mhadi, Zineb Agoumy, Belghait El Hajjaj, Nesma Bendagha, Aida Soufiani, Said Moughil

**Affiliations:** Cardiology A Department, Ibn Sina University Hospital Center, Mohammed V University of Rabat, Rabat, Morocco; Cardiology A Department, Ibn Sina University Hospital Center, Mohammed V University of Rabat, Rabat, Morocco; Cardiology A Department, Ibn Sina University Hospital Center, Mohammed V University of Rabat, Rabat, Morocco; Cardiology A Department, Ibn Sina University Hospital Center, Mohammed V University of Rabat, Rabat, Morocco; Cardiology A Department, Ibn Sina University Hospital Center, Mohammed V University of Rabat, Rabat, Morocco; Cardiovascular Surgery B Department, Ibn Sina University Hospital Center, Mohammed V University, Rabat, Rabat, Morocco

## Abstract

Endomyocardial fibrosis (EMF) is a rare and often underdiagnosed form of restrictive cardiomyopathy. Prognosis is generally unfavorable. Early diagnosis, along with surgical and medical intervention, is crucial for improved outcomes. We report the case of a 15-year-old girl from Guinea who presented with suspected Ebstein’s anomaly and severe right heart failure. Multimodal cardiac imaging revealed right ventricular EMF. Despite counseling on prognosis, the family declined surgery. The patient experienced cardiac arrest two months later.

## CASE DESCRIPTION

A 15-year-old Guinean girl was referred to our cardiology department due to suspected Ebstein’s anomaly presenting with severe right heart failure.

The physical examination revealed a murmur indicative of tricuspid regurgitation and signs consistent with right-sided heart failure.

Her electrocardiogram indicated right atrial hypertrophy.

Transthoracic echocardiography revealed an ectatic right atrium that hindered the analysis of other cardiac chambers, accompanied by signs of diastolic dysfunction on Doppler imaging.

Peripheral blood count didn’t show hypereosinophilia.

**Figure 1:**
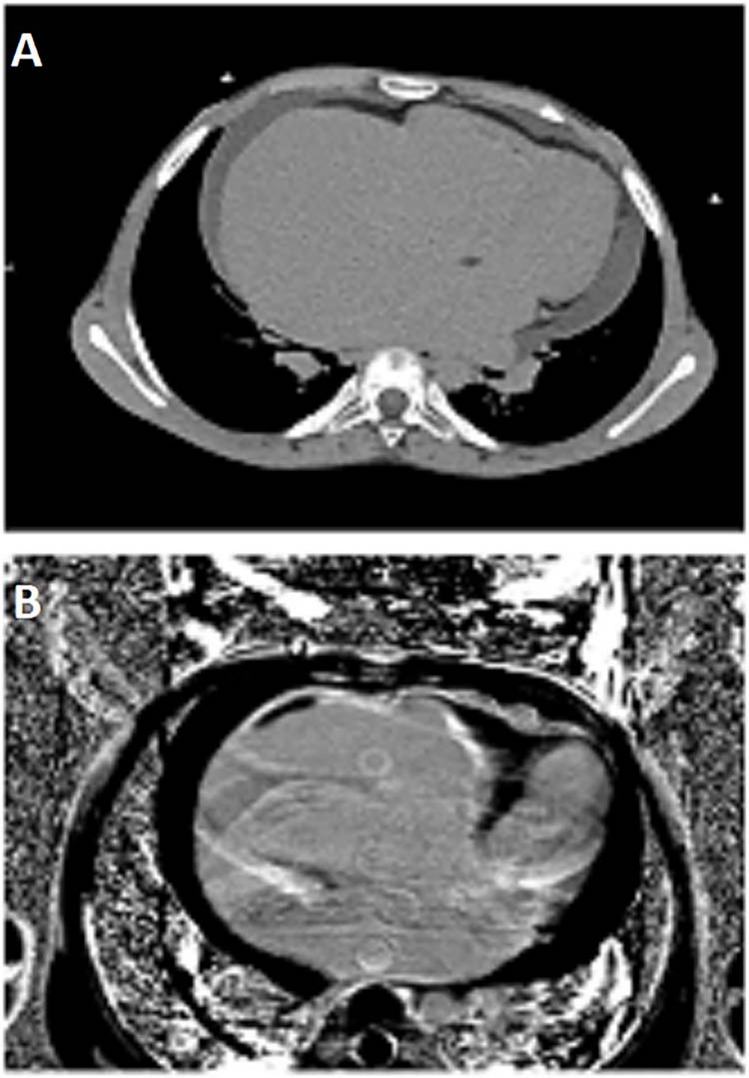
(**A**) Cardiac CT-scan revealing severe dilation of the right atrium associated with thrombosis in right atrium and ventricle. (**B**) CMR: 4-chambers view LGE sequence showing right ventricular endomyocardial fibrosis, with enhancement alongside thrombosis consistent with typical ‘three-layered’ pattern.

Cardiac CT scan demonstrated severe dilation of the right atrium, associated with thrombosis in right atrium and ventricle (Fig. 1A).

Cardiac magnetic resonance (CMR) allowed for the diagnosis of right ventricular EMF with late gadolinium enhancement alongside thrombosis, consistent with typical ‘three-layered’ pattern (Fig. 1B).

The family was informed of the prognosis but declined surgical intervention. The patient was discharged home with oral anticoagulation.

Unfortunately, she experienced a cardiac arrest two months later.

EMF is a rare and often underdiagnosed cause of restrictive cardiomyopathy. The etiology of this condition remains undefined, potentially arising from a convergence of clinical factors interacting with genetic predispositions in individuals susceptible to an inflammatory process leading to fibrotic formation [1].

Suspecting EMF requires attention to signs and symptoms of restrictive heart failure, particularly in endemic regions. Key ECG evidence includes manifestations of right atrial overload. Echocardiographic indicators encompass increased atrial volume, normal ventricular volume, atrioventricular valve dysfunction due to subvalvular fibrosis, and apical obliteration of one or both ventricles [1].

The gold standard for confirming the diagnosis of EMF is CMR, playing a pivotal role in its accurate assessment.

The disease prognosis is generally unfavorable, marked by a high incidence of sudden cardiac death, thromboembolic complications, and end-stage heart failure [2].

Early diagnosis and appropriate management are crucial for enhancing outcomes, involving a combination of medical therapy and surgical intervention [3].
